# Qualitative Exploration of Barriers to Statin Adherence and Lipid Control: A Secondary Analysis of a Randomized Clinical Trial

**DOI:** 10.1001/jamanetworkopen.2021.9211

**Published:** 2021-05-04

**Authors:** Iwan Barankay, Peter P. Reese, Mary E. Putt, Louise B. Russell, Caitlin Phillips, David Pagnotti, Sakshum Chadha, Kehinde O. Oyekanmi, Jiali Yan, Jingsan Zhu, Kevin G. Volpp, Justin T. Clapp

**Affiliations:** 1 Department of Management, The Wharton School, University of Pennsylvania, Philadelphia; 2 Department of Business Economics and Public Policy, The Wharton School, University of Pennsylvania, Philadelphia; 3 Center for Health Incentives and Behavioral Economics, University of Pennsylvania, Philadelphia; 4 Department of Biostatistics, Epidemiology and Informatics, Perelman School of Medicine, University of Pennsylvania, Philadelphia; 5 Division of Renal Electrolyte and Hypertension, Department of Medicine, Perelman School of Medicine, University of Pennsylvania, Philadelphia; 6 Department of Medical Ethics and Health Policy, Perelman School of Medicine, University of Pennsylvania, Philadelphia; 7 Leonard Davis Institute of Health Economics, University of Pennsylvania, Philadelphia; 8 currently a medical student at Rutgers New Jersey Medical School, Newark; 9 Department of Emergency Medicine, Perelman School of Medicine, University of Pennsylvania, Philadelphia; 10 Division of General Internal Medicine, Department of Medicine, Perelman School of Medicine, University of Pennsylvania, Philadelphia; 11 Center for Health Equity Research and Promotion, Cresencz Veterans Affairs Medical Center, Philadelphia, Pennsylvania; 12 Department of Health Care Management, The Wharton School, University of Pennsylvania, Philadelphia; 13 Department of Anesthesiology and Critical Care, Perelman School of Medicine, University of Pennsylvania, Philadelphia

## Abstract

**Importance:**

Financial incentives may improve health by rewarding patients for focusing on present actions—such as medication regimen adherence—that provide longer-term health benefits.

**Objective:**

To identify barriers to improving statin therapy adherence and control of cholesterol levels with financial incentives and insights for the design of future interventions.

**Design, Setting, and Participants:**

This qualitative study involved retrospective interviews with participants in a preplanned secondary analysis of a randomized clinical trial of financial incentives for statin therapy adherence. A total of 636 trial participants from several US insurer or employer populations and an academic health system were rank ordered by change in low-density lipoprotein cholesterol (LDLC) levels. Participants with the most LDLC level improvement (high-improvement group) and those with LDLC levels that did not improve (nonimprovement group) were purposively targeted, stratified across all trial groups, for semistructured telephone interviews that were performed from April 1 to June 30, 2018. Interviews were coded using a team-based, iterative approach. Data were analyzed from July 1, 2018, to October 31, 2020.

**Main Outcomes and Measures:**

The primary outcome was mean change in LDLC level from baseline to 12 months; the secondary outcome, statin therapy adherence during the first 6 months.

**Results:**

A total of 54 patients were interviewed, divided equally between high-improvement and nonimprovement groups, with a mean (SD) age of 43.5 (10.3) years; 36 (66.7%) were women, 28 (51.9%) had diabetes, and 18 (33.3%) had cardiovascular disease. Compared with the high-improvement group, the nonimprovement group had fewer interviewees with an annual income of greater than $50 000 (11 [40.7%] vs 22 [81.5%]), worse self-reported health (fair to poor, 13 [48.1%] vs 3 [11.1%]), more Black interviewees (16 [59.3%] vs 4 [14.8%]), and lower baseline LDLC levels (>160 mg/dL, 2 [7.4%] vs 25 [92.6%]). Participants in the nonimprovement group had a greater burden of chronic illness (≥2 chronic conditions, 13 [48.1%] vs 6 [22.2%]) and were less frequently employed (full-time, 6 [22.2%] vs 12 [44.4%]). In interviews, the nonimprovement group was less focused on risks of high LDLC levels, described less engagement in LDLC level management, articulated fewer specific nutritional choices for optimizing health, and recounted greater difficulty obtaining healthy food. Participants in both groups had difficulty describing the structure of the financial incentives but did recall features of the electronic pill containers used to track adherence and how those containers affected medication routines.

**Conclusions and Relevance:**

Participants in a statin adherence trial whose LDLC levels did not improve found it more difficult to create medication routines and respond to financial incentives in the context of complex living conditions and a high burden of chronic illness. These findings suggest that future studies should be more attentive to socioeconomic circumstances of trial participants.

**Trial Registration:**

ClinicalTrials.gov Identifier: NCT01798784

## Key Points

**Question:**

What barriers to statin therapy adherence and control of cholesterol levels are revealed through qualitative interviews with participants in a randomized trial of financial incentives for adherence?

**Findings:**

In this qualitative study of 54 participants, individuals whose cholesterol levels did not improve described a greater burden of chronic illness, were less frequently employed, were less focused on the risks of high cholesterol levels, appeared to have lower health literacy, were less engaged in their cholesterol level management, made fewer specific nutritional choices for optimizing health, and had greater difficulty obtaining healthy food compared with participants with marked improvement of cholesterol levels.

**Meaning:**

These findings suggest that future interventions should consider addressing socioeconomic circumstances in combination with adherence interventions among patients needing to reduce cholesterol levels.

## Teaser

This qualitative study identifies barriers to improving statin therapy adherence and control of cholesterol levels with financial incentives and insights for the design of future interventions among patients with high cholesterol levels.

## Introduction

Heart disease is the leading cause of death in North America.^1^ Individuals with atherosclerotic cardiovascular disease (ASCVD) can improve their survival by taking statins (hydroxymethyl glutaryl coenzyme A reductase inhibitors).^2^ Among individuals at elevated risk for ASCVD but without prior cardiac events, such as adults with diabetes, the US Preventive Services Task Force^3^ concluded that statins reduce the risk of death and ASCVD. Statins involve manageable adverse effects and are available as low-cost generics.^4,5^ Unfortunately, nonadherence to statin therapy is surprisingly common.^6,7^ Successful and durable strategies are needed to remove barriers to adherence and healthy routines.^3,8,9^ Present bias, whereby individuals undervalue the effect of healthy actions on their future well-being, is a fundamental behavioral barrier to adherence that may be addressed with small targeted incentives rewarding present actions.^10,11^

The Habit Formation trial, a randomized clinical trial described elsewhere,^12,13^ tested the ability of 6 months of financial rewards for daily statin therapy adherence to establish longer-term adherence habits. The primary outcome was change in low-density lipoprotein cholesterol (LDLC) levels at 12 months. The incentives were designed using behavior modification approaches based on insights from economics, management, and psychology.^12,14,15,16,17,18^ Every participant received daily reminders and was asked to use an electronic pill container. Container opening times were transmitted via the cellphone network to track adherence. The trial had a control group as well as 3 incentive groups with equal expected payouts: the first group received daily rewards for adherence; the second group’s incentives were halved if they took their statins after receipt of a daily reminder; and the third group received half their rewards daily and the other half as a monthly deposit that decreased according to the number of days of nonadherence to statin therapy.

Financial incentives make immediate the importance of adherence, which is particularly relevant because statins carry no immediately perceptible health benefits. In addition, incentives may enhance a patient’s ability to focus on establishing lasting daily routines for adherence, which is important because patients at high risk of ASCVD typically need to take statins for life.

It is unknown, however, how best to design and communicate about incentives for medication adherence. In our clinical trial, the mean change in LDLC levels at 12 months was very similar between controls and those receiving daily financial incentives, despite improvements in measured statin therapy adherence recorded through electronic pill-bottle openings among intervention arm participants. In this qualitative study, we used semistructured interviews to compare the experiences of participants with large improvements vs no improvement in LDLC levels during the 12-month study period. This work was consistent with the trial’s secondary aim of exploring socioeconomic backgrounds as a factor in treatment response. We aimed to better understand the variation in effectiveness of incentives between individuals and general barriers to control of cholesterol levels and thereby inform the design of future adherence interventions.^19^

## Methods

### Overview

This study follows the Standards for Reporting Qualitative Research (SRQR) guideline. We applied a mixed-methods sequential explanatory design.^20^ The randomized clinical trial was conducted from August 1, 2013, to July 30, 2018 (the first phase; the trial protocol is found in Supplement 1), with a qualitative interview study among a subset of trial participants from April 1 to June 30, 2018 (the second phase), to illuminate aspects of the quantitative findings from the trial. Investigators are increasingly implementing sequential explanatory designs to help explain the results of randomized clinical trials.^19^ The 4-group randomized clinical trial assessed the efficacy of financial incentives to promote sustained adherence to statin therapy and reduce LDLC levels in individuals at high risk for ASCVD.^12,13^ The qualitative study reported herein enrolled trial participants from Penn Medicine, the largest health care organization serving the Philadelphia region,^21^ and was approved by the University of Pennsylvania institutional review board. All participants provided written informed consent.

As described elsewhere, participants were eligible for the trial if they had a daily statin prescription, self-reported incomplete adherence to the statin therapy, and 1 or more of the following: diabetes, ASCVD with elevated LDLC levels, 10-year ASCVD risk score of greater than 7.5%, or high LDLC levels (>190 mg/dL [to convert to millimoles per liter, multiply by 0.0258]).^12,13,22^ All participants received electronic pill containers, which transmitted a signal to the study team at each opening as a measure of adherence. Participants also received daily reminders to take their statin. Participants had LDLC levels assessed at baseline and 6 and 12 months. The control group received no further intervention. Those in the treatment groups were offered daily financial incentives for adherence, as measured by the pill container, and received daily feedback about their earnings. Participants were paid monthly. Incentives were implemented for 6 months, followed by a 6-month follow-up period without incentives or reminders.

The primary outcome was mean change in LDLC level from baseline to 12 months. The secondary outcome was medication adherence during the first 6 months. On average, all groups saw substantial improvement in LDLC levels. Although financial incentives raised measured adherence in the treatment vs control groups, no significant differences were found between intervention and control groups in LDLC level at 12 months.

### Sampling and Data Collection for the Qualitative Study

We used extreme case sampling (a type of purposive sampling) to select interviewees. As used in the qualitative phase of an explanatory sequential design, extreme sampling targets cases at the outer ends of the distribution along a quantitative parameter of interest (in this case, change in LDLC level from baseline to 12 months). Such cases are assumed to present particularly rich information about that parameter. This sampling strategy facilitates comparison between cases to explore potential causes of stark quantitative differences. We used this sampling approach to examine how the experiences of patients with high LDLC level improvement in the trial differed from those whose LDLC levels improved least.^23^ First, of 805 total trial participants, we rank ordered the 636 participants with LDLC level measurements at both baseline and 12 months according to their change in LDLC levels. We then recruited from 2 groups: (1) the high-improvement group, starting with the participant with the largest decline in LDLC levels and working down the rank-ordered list; and (2) the nonimprovement group, starting with the participant with the largest increase of LDLC levels and working up the rank-ordered list, eventually including some participants with small LDLC level declines. We stratified both groups across the trial’s 4 groups: as we neared code saturation, we began capping the number of interviewees recruited from specific groups, skipping over eligible participants in the LDLC level rank orders if they belonged to a capped group, until we achieved an equal stratification. The overall response rate was 41.9% (54 of 129 participants).

Semistructured interviews were conducted by telephone by 3 team members (C.P., D.P., and S.C., with all research staff who had worked on the randomized clinical trial) and supervised by a faculty anthropologist with substantial experience in qualitative methods (J.T.C.). Recruited participants engaged in audio-recorded, one-on-one interviews (see eMethods in Supplement 2 for interview guide).^24^ Interviews addressed participants’ overall health status, their risk perceptions of high cholesterol levels and any behaviors they had implemented to address it, circumstances of their daily lives, their statin-taking routines, their experiences with financial incentives during the trial, their understandings of how they performed in the trial, and perceived effects of the trial on their long-term behavior. As described below, data were collected until code saturation was achieved, that is, until additional data did not necessitate any alterations to the codebook.^25^

### Qualitative Analysis

A professional service transcribed the recordings. Coding of transcripts was managed with NVivo, version 12 (QSR International) and used an iterative process of codebook development and rounds of double coding to assess reliability and refine the codebook. eMethods in Supplement 2 provides details of this process.

Once coding was complete, 2 investigators (C.P. and D.P.) independently compared the responses of participants in the high-improvement vs nonimprovement groups and of participants in the control group vs those in the combined financial incentives groups. After independently identifying thematic differences along these 2 dimensions, they met to reach consensus on a core set of differences. Finally, a third investigator (J.T.C.) reviewed and verified the differences agreed on by the previous 2 investigators. After coding and comparison were complete, the observed differences were explored via regular group discussion using an abductive approach, which is designed to facilitate explanation of findings that are not well explained by extant literature on a topic.^26,27^ We identified potential explanations for unexpected thematic trends, further evaluating them against the data to assess their level of empirical support until we arrived at an account that best reflected the data.

### Statistical Analysis

Data were analyzed from July 1, 2018, to October 31, 2020. Characteristics of interviewees and trial participants were described at baseline and 12 months using percentages and 95% CIs.

## Results

Table 1 displays the 54 interviewees’ characteristics (36 women [66.7%] and 18 men [33.3%]; mean [SD] age, 43.5 [10.3] years) by improvement group. Twenty-eight interviewees (51.9%) had diabetes, and 18 (33.3%) had cardiovascular disease. At baseline, 25 of 27 interviewees (92.6%) in the high-improvement group had highly elevated LDLC levels (>160 mg/dL) vs 2 of 27 (7.4%) in the nonimprovement group. By 12 months, the distribution had changed notably: 16 of the high-improvement interviewees (59.3%) had LDLC levels of less than 100 mg/dL, whereas 11 of the nonimprovement group interviewees (40.7%) had LDLC levels of greater than 160 mg/dL. Interviewees in the high-improvement group achieved a mean LDLC level decrease of 122 mg/dL compared with a mean increase of 33 mg/dL in the nonimprovement group and had higher statin therapy adherence during 6 months (standardized mean difference, 0.313).

**Table 1.** Demographic and Clinical Characteristics of Study Participants

Characteristic	Interview group^a^	
	High-improvement (n = 27)	Nonimprovement (n = 27)
Age, y		
<35	0	0
35-49	5 (18.5)	4 (14.8)
50-59	12 (44.4)	8 (29.6)
60-69	5 (18.5)	12 (44.4)
≥70	5 (18.5)	3 (11.1)
Female	17 (63.0)	19 (70.4)
Male	10 (37.0)	8 (29.6)
Race		
Black	4 (14.8)	16 (59.3)
White	20 (74.1)	11 (40.7)
Other	3 (11.1)	0
Annual income, USD		
<50 000	5 (18.5)	16 (59.3)
≥50 000	22 (81.5)	11 (40.7)
Baseline LDLC level, mg/dL		
100-129	0	19 (70.4)
130-159	2 (7.4)	6 (22.2)
160-189	2 (7.4)	1 (3.7)
≥190	23 (85.2)	1 (3.7)
12-mo LDLC level, mg/dL		
<100	16 (59.3)	0
100-129	7 (25.9)	2 (7.4)
130-159	4 (14.8)	14 (51.9)
160-189	0	6 (22.2)
≥190	0	5 (18.5)
Health condition		
Excellent	1 (3.7)	0
Very good	8 (29.6)	7 (25.9)
Good	15 (55.6)	7 (25.9)
Fair	2 (7.4)	12 (44.4)
Poor	1 (3.7)	1 (3.7)
Cardiovascular disease	5 (18.5)	13 (48.1)
Diabetes	7 (25.9)	21 (77.8)
Measured adherence, mean (95% CI)		
During first 6 mo	0.82 (0.72-0.92)	0.70 (0.59-0.81)
During last 30 d of intervention	0.83 (0.73-0.93)	0.57 (0.40-0.73)

^a^ Unless otherwise indicated, data are expressed as number (percentage) of participants. Percentages have been rounded and may not total 100. Improvement refers to decrease in LDLC level from baseline to 12-month follow-up in the randomized clinical trial.

The Figure depicts recruitment for this qualitative study. We interviewed 54 individuals equally divided between high-improvement and nonimprovement groups. Each group included 12 control and 15 treatment participants, stratified equally across the 3 incentive groups.

**Figure.**
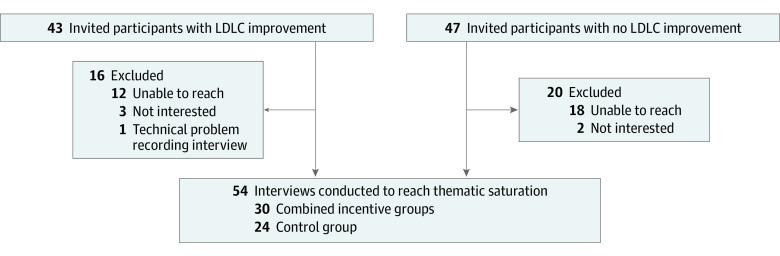
Recruitment Flowchart Staff contacted participants following a rank-order approach until all stratification cells were complete. Incentive groups were stratified equally across the 3 incentive groups by interview group. LDLC indicates low-density lipoprotein cholesterol.

There were important socioeconomic and health differences between the groups. Ten interviewees in the high-improvement group (37.0%) were 60 years or older, 4 (14.8%) were Black, and 22 (81.5%) had incomes of $50 000 or more, compared with 15 (55.6%) who were 60 years or older, 16 (59.3%) who were Black, and 11 (40.7%) with incomes of $50 000 or more in the nonimprovement group. Three interviewees in the high-improvement group (11.1%) reported fair or poor health compared with 13 in the nonimprovement group (48.1%). Last, although some individuals in the high-improvement group had cardiovascular disease (5 [18.5%]) or diabetes (7 [25.9%]), almost half of the nonimprovement group (13 [48.1%]) had cardiovascular disease, and more than three-quarters (21 [77.8%]) had diabetes. These differences were consistent with those of the eligible pool of interviewees in the top and bottom deciles of LDLC level change, suggesting that our sample is representative based on observable characteristics (Table 2).

**Table 2.** Demographic and Clinical Characteristics of Participants in the Randomized Clinical Trial With the Highest and Lowest Deciles of LDLC Level Change During the Trial

Characteristic	Eligible based on trial deciles (n = 129)		
	Improvement^a^		Difference, % (95% CI)
	Highest (n = 65)	Lowest (n = 64)	
Baseline LDLC level, mg/dL			
100-129	0	43 (67.2)	−67.2 (−78.7 to −55.7)
130-159	3 (4.6)	16 (25.0)	−20.4 (−32.2 to −8.6)
160-189	6 (9.2)	2 (3.1)	6.1 (−2.1 to 14.3)
≥190	56 (86.2)	3 (4.7)	81.5 (71.6 to 91.3)
12-mo LDLC level, mg/dL			
<100	35 (53.8)	0	53.8 (41.7 to 66.0)
100-129	21 (32.3)	12 (18.8)	13.6 (−1.3 to 28.4)
130-159	7 (10.8)	26 (40.6)	−29.9 (−44.1 to −15.7)
160-189	1 (1.5)	15 (23.4)	−21.9 (−32.7 to −11.1)
≥190	1 (1.5)	11 (17.2)	−15.6 (−25.4 to −5.9)
Age, y			
<35	2 (3.1)	1 (1.6)	1.5 (−3.7 to 6.7)
35-50	20 (30.8)	12 (18.8)	12.0 (−2.7 to 26.8)
50-60	21 (32.3)	26 (40.6)	−8.3 (−24.9 to 8.2)
60-70	13 (20.0)	18 (28.1)	−8.1 (−22.8 to 6.6)
≥70	9 (13.8)	7 (10.9)	2.9 (−8.4 to 14.3)
Female	37 (56.9)	48 (75.0)	−18.1 (−34.1 to −2.0)
Male	28 (43.1)	16 (25.0)	18.1 (2.0 to 34.1)
Race			
Black	20 (30.8)	38 (59.4)	−28.6 (−45.1 to −12.2)
White	38 (58.5)	22 (34.4)	24.1 (7.4 to 40.8)
Other	7 (10.8)	4 (6.3)	4.5 (−5.1 to 14.1)
Income, USD			
<50 000	19 (29.2)	40 (62.5)	−33.3 (−49.5 to −17.1)
≥50 000	44 (67.7)	24 (37.5)	30.2 (13.8 to 46.6)
Do not wish to answer	2 (3.1)	0	3.1 (−1.1 to 7.3)
Health condition			
Excellent	3 (4.6)	2 (3.1)	1.5 (−5.2 to 8.1)
Very good	20 (30.8)	13 (20.3)	10.5 (−4.5 to 25.4)
Good	33 (50.8)	22 (34.4)	16.4 (−0.4 to 33.2)
Fair	8 (12.3)	25 (39.1)	−26.8 (−41.1 to −12.4)
Poor	1 (1.5)	2 (3.1)	−1.6 (−6.8 to 3.6)
Cardiovascular disease	10 (15.4)	24 (37.5)	−22.1 (−36.9 to −7.4)
Diabetes	16 (24.6)	48 (75.0)	−50.4 (−65.3 to −35.5)
Adherence, mean (95% CI)^b^			
6 mo of intervention	0.84 (0.78 to 0.89)	0.79 (0.74 to 0.84)	0.218 (−0.128 to 0.564)
During last 30 d of intervention	0.80 (0.74 to 0.87)	0.71 (0.63 to 0.79)	0.313 (−0.034 to 0.660)

Abbreviations: LDLC, low-density lipoprotein cholesterol; USD, US dollars.

^a^ Unless otherwise indicated, data are expressed as number (percentage) of participants. Percentages have been rounded and may not total 100. Improvement refers to decrease in LDLC level from baseline to 12-month follow-up in the randomized clinical trial.

^b^ Standardized mean differences were calculated for continuous variables instead of percentage differences.

### Overall Health, Function, and Employment Status Reported in Interviews

Six interviewees in the high-improvement group (22.2%) reported at least 2 chronic conditions compared with 13 in the nonimprovement group (48.1%). This greater burden of chronic illness accords with the lower self-reported health status in the nonimprovement group (Table 1). Despite these differences, interviewees from both groups described similar levels of functional limitation and dependency. However, individuals in the high-improvement group were more likely to work: 12 of 27 (44.4%) were full-time employees, 5 (18.5%) were part-time employees, 2 (7.4%) were retirees, and none were disability beneficiaries. In comparison, only 6 interviewees in the nonimprovement group (22.2%) reported working full-time, 1 (3.7%) worked part time, 2 (7.4%) were retired, and 3 (11.1%) received disability benefits.

### Recollection of Financial Incentives

During the trial’s 6-month intervention period, intervention group participants received incentives for adherence and daily feedback about earnings. However, few interviewees who were enrolled in the study’s incentives groups, irrespective of improvement status, could describe the structure of rewards (see Table 3, section 1). Some participants did not distinguish between incentives for adherence and payments for study participation; as 1 interviewee in the nonimprovement group stated, “All I know is they paid me some for every so many months or something.”

**Table 3.** Understanding of Intervention Design

Participant understanding	Illustrative questions and answers
Section 1: Participants have difficulty differentiating financial rewards from study payments	Interviewer: Were you eligible to receive financial rewards if you took your medication regularly?
	High-improvement interviewee: Was I able to? Yes.
	Interviewer: And can you recall how those bonuses worked?
	High-improvement interviewee: I think it was when I first started and then because I had to go for blood work, and I think it was every time I went for blood work, and then maybe at the end of the study, I got—
	Interviewer: And did you receive any rewards for taking your medication regularly?
	High-improvement interviewee: No. I don’t recall that.
	Nonimprovement interviewee: All I know is they paid me some for every so many months or something.
	Interviewer: Were you eligible to receive financial rewards if you took your medication regularly?
	High-improvement interviewee: I believe so.
	Interviewer: And can you recall how these bonuses worked?
	High-improvement interviewee: No. I know that I received milestone payments, I think for completion. Maybe I got points along the way for number of compliance days. I’m not sure. Sorry.
	Interviewer: Can you recall were you eligible to receive financial rewards if you took your medicine regularly?
	Nonimprovement interviewee: Well, I think during the study, sometime I think it, a couple times, extra money for doing something, but I can’t remember what it was.
Section 2: Participants recall details of wireless pillbox	Interviewer: And then throughout the study, we would have asked you to store your cholesterol medicine in an electronic pill bottle. Do you remember that pill bottle?
	Response: Yeah, I just got rid of it. I kept it all that time. I just got rid of it.… But yes, I kept it in that bottle all the time.… It was like a square. It wasn’t round. But it looked round, but yet it was square and it had a round top and it had a light that went around the bottom of it, so when you opened the top, the bottom of it lit up.
	Interviewer: [C]an you recall what type of pill bottle you used during this study?
	Response: It was—I think I still have it. It was a pill bottle where I plugged a device into the wall and every time I unscrewed the top on it, it would send, I guess, a signal to y’all. Every time I unscrewed the cap and took it.
Section 3: High improvers rarely motivated by incentives	Interviewer: Did you find that the financial incentives were a major motivating factor for taking your statin medication?
	High-improvement interviewee: No.
	Interviewer: Why not?
	High-improvement interviewee: Why? Because my cholesterol was so high I had to do something.
	Interviewer: Okay. How much greater would the financial rewards have to be in order to make a difference in how regularly you take your statin medication?
	High-improvement interviewee: None. I’m going to take it for the rest of my life.
	High-improvement interviewee: I don’t think that there would have been a monetary amount that would have made a difference. Seeing my test results and feeling better made a difference. Yeah.
	Interviewer: So how much greater would the rewards have to be in order to make a difference in how regularly you took your statin medication?
	High-improvement interviewee: I don’t know that there’s a correlation between the two. I mean, it was not a prime motivator for me.
	Interviewer: And what aspect of the trial do you think was most helpful to getting you to regularly take your statin medication?
	High-improvement interviewee: My participation in the study sort of created the sense that somebody was checking up on me, that it was being monitored to some degree. And that was an incentive to do better at taking the medication on a regular basis.
Section 4: Low improvers motivated by incentives	Low-improvement interviewee: It’s like, oh, if I do this, then I’m gonna get something in return, or at least get a chance at something in return. So if it said, okay, if you took your medicine today your name goes into the drawing or whatever. And so to me, that made me think about taking the medicine more often.
	Low-improvement interviewee: I’m constantly looking for opportunities, as single seniors are. I don’t have a big pension and I can’t live in subsidized housing, and I’m like in between a rock and a hard place with the income.... So unless somebody asks me to take an experimental drug, I’m eager to participate in studies.... The financial motivation was an added incentive.
	Low-improvement interviewee: It helped. See, the money wasn’t every month. So I don’t even remember what it was. But every little bit helps you. You got a nickel and dime to get what you need. Survival.
	Low-improvement interviewee: I know I was getting checks and they were good. They helped me a lot. I was surprised when I would see a check, and it helped me a lot.

In contrast, those in the incentive groups in particular offered detailed recollections of the features of the electronic pill bottles. Recalled 1 trial participant, “[the pill bottle] wasn’t round. But it looked round, but yet it was square and it had a round top and it had a light that went around the bottom of it, so when you opened the top, the bottom of it lit up” (Table 3, section 2). Of note, no interviewees expressed concern about their adherence being monitored through the pill bottle.

Interviewees were asked about the effect of the study earnings on their financial situations and pill-taking habits. Twenty-five of the 27 interviewees in the high-improvement group (92.6%) said that their study earnings did not change their regular spending habits, compared 15 of the 27 in the nonimprovement group (55.6%).

Most respondents in the high-improvement group did not find study earnings a substantial motivator to take their statin (Table 3, section 3), explaining that they joined the study to get their cholesterol levels under control, which was a reward in itself. “I don’t think that there would have been a monetary amount that would have made a difference,” said a high-improvement participant. “Seeing my test results and feeling better made a difference.” However, interviewees in the nonimprovement group were more likely to characterize study earnings as a motivator (Table 3, section 4).

### Understanding and Prioritization of LDLC Level Management

When articulating their concerns about having high LDLC levels, the language and viewpoints of the groups differed. These differences may point to discrepancies in health literacy and patient engagement between groups. Interviewees in the high-improvement group universally described their concerns in terms of pathology, often using medicalized language in noting that they worried about “stroke,” “heart disease,” “heart failure,” “plaque buildup,” “cardiovascular” problems, or exacerbation of chronic medical conditions, including some unrelated to LDLC level.

Many interviewees in the nonimprovement group also used medical terms to articulate disease-centered concerns, but their responses were otherwise more varied. Unlike those in the high-improvement group, several interviewees said they currently were not concerned about their LDLC level or had only become concerned after being warned by a health professional that their cholesterol levels were worrisome. Use of colloquial language (eg, “clogged” arteries) to describe pathology was more frequent among interviewees in the nonimprovement group.

When asked about family history of cholesterol issues, 14 interviewees in the nonimprovement group (51.9%) and 15 in the high-improvement group (55.6%) responded affirmatively. When further asked how their family history affected their approach to their own LDLC levels, interviewees in the high-improvement group more frequently stated that it inspired them to alter their health behaviors, with several specifically mentioning medication adherence.

### Dietary and Medication Habits

Respondents in the high-improvement group more frequently mentioned specific nutritional changes they made to improve their LDLC levels than did those from the nonimprovement group (Table 4, section 1). Six of 7 employed interviewees in the nonimprovement group cited a lack of access to healthy foods in their work environments or an inability to find time to eat healthy foods due to hectic work schedules (Table 4, section 2). Conveyed 1 interviewee in the nonimprovement group: “I didn’t exactly live the greatest of lifestyle[s]. I worked shift work almost my entire life. When you’re working at night the only place open to eat at is WaWa, a gas station, or there was a diner that was in town.” In contrast, just 3 of the 17 employed interviewees in the high-improvement group described such difficulties.

**Table 4.** Dietary and Medication Habits Entering the Trial

Participant habit	Illustrative questions and responses
Section 1: High-level improvers make more specific dietary changes	Interviewer: [P]rior to your participation in our study, were you doing anything else to try and lower your cholesterol?
	Nonimprovement interviewee: No. Oh, eggs. I cut out eggs.
	Interviewer: …Did you change your diet in any other way?
	Nonimprovement interviewee: No. I try to eat diet food, but it don’t work for me.
	Interviewer: Okay. And by cutting out eggs, were you able to successfully lower your cholesterol?
	Nonimprovement interviewee: No. No, it’s still high.
	Interviewer: [P]rior to your participation in our study, were you doing anything else to try to lower your cholesterol?
	Nonimprovement interviewee: Not that I can remember. I mean I can’t remember anything different.
	Interviewer: …And were you able to successfully lower your cholesterol before the study?
	Nonimprovement interviewee: Not before the study, no. I mean the doctor helped me along with that with telling me what I was doing wrong with eating and so he—the doctor helped me along with it.
	Interviewer: And did you find those modifications to your normal lifestyle, like the diet and exercise, were they challenging?
	Nonimprovement interviewee: Yes, they were very challenging to me.… [H]ow to eat was the biggest change in my life. How to eat better was the biggest change because I was eating anything I wanted to.
	High-improvement interviewee: I was taking fish oil, trying to watch what I eat. I don’t eat any fish, so that’s always been an issue. And that was really about it, and I couldn’t get it down. I mean, no matter what I ate, it was still high. So I think it’s more of a familial cholesterol.…
	Interviewer: [B]efore the study you were taking fish oil, watching what you were eating. Was that challenging?
	High-improvement interviewee: Yes. Because all the good stuff has bad stuff in it—butter, all of that kind of stuff. But you read—you try to read online and you can reach for articles that tell you plenty of things about—I did see a dietician while I was there. It kind of helped me a little bit. It brought it down a tenth, so that was helpful.
Section 2: Nonimprovers have difficulty accessing healthy food while working	Nonimprovement interviewee: I fluctuate my diet, sometimes I’m good, sometimes I’m bad. That’s probably why I’m still a diabetic.… It’s just in the family and my concern with the cholesterol is that it’s gonna affect my heart. I didn’t exactly live the greatest of lifestyle[s]. I worked shift work almost my entire life. When you’re working at night the only place open to eat at is WaWa, a gas station, or there was a diner that was in town.
	Nonimprovement interviewee: Well, it’s always hard to change diet habits, especially when you work in areas that people are constantly bringing not so heart-healthy food around. You know, it’s just a challenge in general to change your diet completely.
	Nonimprovement interviewee: I’m a teacher. And so it’s very hard during the day. You have 30 minutes for lunch. So unless you’re really, really, really very prepared in advance it’s really hard to find foods that kinda fit into the mold of what you’re trying to do. The cafeteria doesn’t offer anything to me that’s even a little bit remotely healthy for our students, much less trying to meet dietary needs. So it was definitely a struggle.
Section 3: Nonimprovers lack preestablished pill-taking regimens	Interviewer: [B]efore participating in our study what was your daily routine like for taking your statin medication?
	Nonimprovement interviewee: There really wasn’t much of a routine. I kinda forgot it most of the time.
	Interviewer: Okay. So did you organize your pills in any way or use any device?
	Nonimprovement interviewee: No.
	Interviewer: [B]efore participating in our study what was your routine for taking your cholesterol medicine? For example, do you have a pill organizer?
	Nonimprovement interviewee: No. I just knew which ones to take and I took the cholesterol pill at night, so there was no pill box or anything like that. I know when to take—my high blood pressure medicine, I would take in the morning.
	Interviewer: Okay. Where did you normally keep your cholesterol medicine?
	Nonimprovement interviewee: In the bathroom in the medicine cabinet.
	Interviewer: Would you say that you took your cholesterol medicine regularly prior to participation in our study?
	Nonimprovement interviewee: No. I didn’t take it regularly. No.
	Interviewer: So before participation in our study, what was your routine for taking your statin medication?
	Nonimprovement interviewee: I don’t even—I can’t even remember. I really wasn’t doing anything, just sitting around getting fat. When you retire, you can do things like that.… I would forget to take it if it’s not on my mind. If I took it, I took it. Not really, because I forgot. But now, I take it more regular.
Section 4: Study pill bottle does not enhance preestablished routines of high improvers	High-improvement interviewee: I’ll tell you that [using the study pill bottle] was harder to remember to take than the way I’ve always taken it.… I would have to remember to go to 2 places to take all my meds.… [I]t was even harder for me to remember with that bottle because I just have all of my evening meds in 1 place, and I take them every night without a problem.
	High-improvement interviewee: I hate to say it, but I don’t believe the container or the little gizmo on the wall did anything at all to increase my habit of taking it every day. I think that was pretty much set before and was throughout and remains after. I hate to say that—because I know you guys are doing the study to see how effective it is. I’m not sure in my case it had any effect at all on the regularity with which I took it. I was not in a habit of skipping it, and I never needed any kind of reminder to—oh, yeah, don’t forget to take your medication.… [I] t was a routine that I did regardless of having [the study pill bottle] or not, so I don’t think it had any effect at all, frankly.

When asked about pill-taking routines before the study, interviewees in the high-improvement group largely characterized their pill-taking routine as well established, whereas those in the nonimprovement group more often described themselves as forgetful and unable to establish medication routines (Table 4, section 3). Discussing pill-taking behavior before the trial, a participant in the nonimprovement group recalled, “I can’t even remember. I really wasn’t doing anything, just sitting around getting fat.… I would forget to take it if it’s not on my mind. If I took it, I took it. Not really, because I forgot.” This difference carried through interviewees’ responses about which aspects of the trial were unhelpful. Eight interviewees in the high-improvement group cited the specialized pill bottle used in the trial, which they said interfered with their preestablished routines. An interviewee in the high-improvement group stated: “I’ll tell you that [using the study pill bottle] was harder to remember to take than the way I’ve always taken it.… I would have to remember to go to 2 places to take all my meds.… [I]t was even harder for me to remember with that bottle because I just have all of my evening meds in one place, and I take them every night without a problem.” In contrast, few interviewees in the nonimprovement group reported any aspect of the trial as particularly troublesome.

## Discussion

Medication nonadherence is a major obstacle to achieving gains in health from safe and effective therapies such as statins. The high prevalence of medication nonadherence among patients with ASCVD and other diseases has motivated research to identify adherence barriers and strategies to overcome them.^28,29^ Behavioral economics points to the role of present bias, because the benefits of adherence are often delayed and may seem too far away and abstract to motivate patients.^30,31^ Present bias provides the rationale for trials of small financial incentives that provide immediate benefits for adherence and, if successful, prevent future deterioration in health and associated regret.^12,32^

This qualitative study of our trial of financial incentives for statin adherence provides important insights into the context and living conditions of study participants that may have prevented the incentives from enabling a more substantial improvement in adherence routines.^33^ The differences found between high-improvement and nonimprovement groups suggest the influence of socioeconomic status on participants’ efforts to lower LDLC levels, consistent with prior research on medication adherence.^34,35,36,37,38,39,40,41^ Socioeconomic status is a complex collection of factors, including income, wealth, educational attainment, occupation, location, and social capital. Participants in the high-improvement group were more likely to be employed, evinced greater health literacy and engagement, had better access to healthy food at work and work schedules more amenable to healthy eating, were more able to make specific dietary changes, and had less difficulty establishing medication routines. They were also more likely to have an annual income of $50 000 or greater, to identify as White, to have lower rates of cardiovascular disease and diabetes, and to have self-reported better overall health yet with higher baseline LDLC levels. The same differences appeared when we compared the top and bottom deciles of LDLC level improvement among all participants in the underlying clinical trial, suggesting that the interviewees were representative of these groups.

Our results indicate that individuals of lower socioeconomic status might face distinct challenges in responding to incentives and feedback. Specifically, the physical, logistical, cognitive, and emotional burdens of low socioeconomic status may have made it harder for these individuals to initiate new routines, manage their health, and engage with the study intervention. Even simple changes, such as taking a statin daily, appear to be difficult to initiate in this population, although they were well informed about their medication because they had existing prescriptions and access to routine care. More generous incentives might have been more salient for behavior modification and should be explored in future research, although higher incentives might not be cost-effective.

Our interviews also revealed important aspects of patients’ pill-taking routines and the challenges that remote monitoring via electronic pill bottles may present. In this and a growing number of other trials, remote monitoring of medication adherence is the platform for delivering financial incentives, feedback, and other interventions.^42^ Many individuals in the high-improvement group reported that the pill bottles disrupted their medication routines, whereas the nonimprovement group did not report this problem. Instead, those in the nonimprovement group reported that the device and incentives were not sufficient by themselves to change their routines. Future studies should pay close attention to how electronic pill containers interact with participants’ established routines and provide coaching and health education to those who have clinical inertia and struggle to engage with interventions such as incentives and feedback.

Although study participants were able to provide details about the electronic pill containers, most participants from the incentive groups had little memory of how and which behavior was financially rewarded. This finding stands in contrast to the common assumption that with proper incentives, it is in the interest of participants to understand rewards and how to qualify for them. It emphasizes the potential need to accompany these interventions with multipronged approaches addressing structural barriers to cholesterol level control (such as better access to healthy food and community engagement), as well as customized, motivational interviewing and health education, especially when participants do not show improvement. As noted, compared with a control condition, participants with incentives had significantly higher measured adherence but no difference in LDLC level change. Taken together, these findings and the qualitative data suggest that financial rewards for adherence led some participants to focus attention on the pill container but did not cause participants to remember the incentive structure itself. Follow-up studies need to explore what the barriers to comprehension are and how to overcome them to improve the design of interventions.

### Limitations

This study has limitations. Interviewees’ ability to recall the details of the trial and their experiences participating in it may have been limited because the interviews were conducted more than 6 months after the incentives ended. The response rate was 41.9%; however, qualitative participants were comparable to eligible participants based on observable characteristics. Furthermore, in comparing the experiences of interviewees in the high-improvement and nonimprovement groups, we used 12-month change in LDLC level as a proxy for how successful participants were in the trial. Because the high-improvement group had significantly higher baseline LDLC levels, their much larger decrease in LDLC levels may have in part been owing to their simply having greater room for improvement. However, we affirm that the contrast between groups was valid, because the LDLC levels of most participants in the nonimprovement group actually increased (in stark contrast to the mean decline of >32 mg/dL in the overall trial). Furthermore, in light of the substantial experiential and demographic differences between the groups, we do not believe that the difference in baseline LDLC levels can be posited as the primary explanation of why the groups achieved such divergent outcomes.

## Conclusions

This qualitative study suggests the need for a new approach to the provision of financial incentives. Whereas incentives theories assume that it is in the interest of individuals to manage changes autonomously to qualify for the highest financial rewards, this study highlights that participants with socioeconomic disadvantages and clinical burdens may need a more customized approach along with additional support to address, where possible, structural barriers to improving health.

## Notes

Supplement 1. **Trial Protocol**

Supplement 2. **eMethods.** Qualitative Assessment
