# Supplementary material for: Qualitative Exploration of Barriers to Statin Adherence and Lipid Control: A Secondary Analysis of a Randomized Clinical Trial
Source: JAMA Netw Open. 2021 May 4;4(5):e219211. doi: 10.1001/jamanetworkopen.2021.9211 (PMC8097500; doi:10.1001/jamanetworkopen.2021.9211)
Supplement: Supplement 2. — eMethods. Qualitative Assessment [file jamanetwopen-e219211-s002.pdf]

Supplementary Online Content

Barankay I, Reese PP, Putt ME, et al. Qualitative exploration of barriers to statin adherence and lipid control: a secondary analysis of a randomized clinical trial. *JAMA Netw Open*. 2021;4(5):e219211. doi:10.1001/jamanetworkopen.2021.9211

eMethods. Qualitative Assessment

This supplementary material has been provided by the authors to give readers additional information about their work.

© 2021 Barankay I et al. *JAMA Network Open*.

eMethods. Qualitative Assessment

1.1. Qualitative Assessment Voicemail Message Script

Voicemail message (unidentified): Hi, this message is for (participant's name). This is (CRC name), calling from Penn Medicine to follow-up on a study you recently participated in. If you could please give me a call back my number is 215-573-4224. If I don't answer please leave your name, number, and a good time to call you back. Thank you and have a nice day!

Voicemail message (identified): Hi, this message is for (participant's name). This is (CRC name), calling from Penn Medicine to follow-up on the Way to Healthy Cholesterol study you recently participated in. If you could please give me a call back my number is 215-573-4224. If I don't answer please leave your name, number, and a good time to call you back. Thank you and have a nice day!

1. Hello. My name is (*name*). I am calling from the University of Pennsylvania on behalf of the Way to Healthy Cholesterol study team. Is (*participant name*) available?
    a. IF NOT AVAILABLE→ When would be a good time to call back?

Hi (*participant name*) I am calling today to follow-up on your participation in the Penn Healthy Hearts study from (enrollment date to end of program date). This was the program that involved electronic pill bottles for your statin medication. Thank you for participating!
I am calling to see if you would be willing to participate in an interview about your experience in the study. We have interesting study results and would appreciate your help in better understanding the results and learning more about how people take their statin medications so that we can help other patients.

The interview will take about 20-30 minutes. You will be paid $50 for completing the interview. Your participation is completely voluntary and all of your answers will be kept confidential. Your participation will not affect your present or future care at Penn Medicine.

Do you have any questions?

Obtain and record on this form verbal consent:

Are they interested in proceeding with the phone interview? YES/NO

If YES → Is it ok if I tape record our conversation? This is only to ensure that we capture everything we discuss. Thank you for agreeing to that.

If NO (not interested) → Thank you for your time. Have a good day.

If NO (*not a good time*) → When would be a good time to call back? Should I call back on this number, or is there a different phone number that is best to reach you?

© 2021 Barankay I et al. *JAMA Network Open*.

1.2. Qualitative Assessment Instructions Script

The script below will be read to the subject over the phone before beginning the interview

Study Participation

- Your participation involves participating in a 30-minute, telephone interview. All study activities will take place at a time that is convenient for you.
- The interview will be audio-recorded so that we do not miss anything important that you say. When the audio is being transcribed (typed out), all identifying information, including any names or institutions you mention in the interview, will be removed from the transcript. Therefore, no one reading the transcript of the interview will know who you are.
- If at any point during the interviews you feel uncomfortable or you need to stop for any reason, please let the interviewer know. You can choose to skip any question you do not wish to answer or end the interview at any time if you decide you no longer wish to participate.
- As a participant in this study, you will remain anonymous and all your responses will be de-identified.

Risks and Benefits of Participation

- There is little risk associated with completing an interview. Because we are collecting your name and contact information, there is a small risk that confidentiality and anonymity will be lost, but every precaution is taken to protect this information.

Questions

- If you have any questions about this information, you may contact the study staff. You may also contact the Office of Regulatory Affairs at the University of Pennsylvania with any questions or concerns.
- Would you like me to email you our contact information? Can you provide your email address?

Is now a good time to begin or would you like to schedule another time to complete the survey?

Ok great, let's begin the interview.

© 2021 Barankay I et al. *JAMA Network Open*.

1.3. Qualitative Assessment Interview Script

1. *Overall health status*
    a. Besides high cholesterol, could you describe any other health conditions you have been dealing with recently?
        i. What do you do to treat or manage these conditions?
        ii. How do these conditions affect your daily life?
            1. Do they give you pain or other symptoms?
            2. Do they limit the activities you can do, and if so, how?
    b. In comparison to these other conditions, how much does [OR did] your high cholesterol affect your daily life?

2. *Perception of cholesterol*
    a. What are your concerns, if any, about having high cholesterol?
    b. Do you have a family history of high cholesterol?
        i. How does this history [OR lack of history] affect the way you think about cholesterol?
    c. Prior to your participation in our study, were you doing anything else to try and lower your cholesterol? [altering diet, exercising]
        i. [If yes] Were you able to successfully lower your cholesterol?
        ii. [If yes] Did you find these modifications to your normal lifestyle challenging, and if so, how?
        iii. [If no] Why didn't you end up trying these changes?

3. *Daily life*
    a. What is your schedule like on a typical day?
    b. Do you work?
        i. [If yes] What type of work do you do, and what are your hours like?
    c. Do you have family members or friends that you take care of? [children, etc.]
        i. What do you do each day to take care of these individuals?
        ii. Does anyone help you with these responsibilities?
    d. Do you have a caregiver that helps you with your daily tasks? [cooking, cleaning, transportation, etc.]
    e. Have you found it easy or difficult to fit in your statin medication each day?
        i. What makes it [easy OR difficult]?
    f. Participation in this study required getting your cholesterol tested every six months. Did you find it easy or difficult to get your study labs done?
        i. What made it [easy OR difficult]?

4. *Pill-taking routine*
    a. Before participation in our study, what was your routine for taking your statin medication?
        i. How did you organize your pills? Did you use a device for doing so?
        ii. Where in your home did you keep this device?
        iii. Would you say that you took your statin medication regularly prior to participation in our study?
    b. What type of pill bottle did you use during the study?
        i. Did you find the light on the device helpful?
        ii. What was your process for loading up the device?
        iii. Where did you keep the device?
        iv. Did the device give you any problems during the study?
        v. Do you still use the device?
        vi. How did your routine for taking your statin medication with the study device compare to your routine before the trial? Same/different?
    c. Were there times during the trial when you took your statin medication but not using the study device?

5. *Financial incentives*
    a. How comfortable do you feel about your overall financial situation?
        i. Does anyone help you with your financial responsibilities?

© 2021 Barankay I et al. *JAMA Network Open*.

b. How much money did you earn from the study?
    i. What did you do with this money when you received it?
        1. Did it alter your normal spending patterns?
    ii. Were you eligible to receive financial rewards if you took your medication regularly?
        1. Can you recall how these bonuses worked?
    iii. If participant wants to know how much money they earned from study:
        1. Tell them the standard amount they would have received for enrollment and completion of labs
c. Did you find that the financial incentives were a major motivating factor for taking your statin medication?
    i. [If no] Why not?
    ii. [If no] How much greater would the financial rewards have to be in order to make a difference in how regularly you take your statin medication?

6. *Study performance*
    a. Approximately what was your baseline LDL ('bad cholesterol') when you were first tested as part of our study?
    b. Did your LDL go up or down over the course of our study?
        i. What was your reaction to getting those results? Did you change how often you took your medication or change any other aspects of your life?
    c. During the trial, did you do anything else other than taking your statin medication to try and lower your LDL?
        i. [If yes] Did you make any changes to your exercise?
        ii. [If yes] Did you make any changes to your diet?
        iii. [If yes] Did you take any other medications, or supplements?
        iv. [If yes] Were you also doing these things before the trial?
            1. [If not doing before trial] Did participation in the trial encourage you to do these things?

7. *Durability*
    a. How were you reminded to take your statin medication during the study?
        i. What role did these reminders play in whether you took your medication or not? [How did you use these reminders?]
        ii. Do you think the reminders were helpful? Why or why not?
    b. Do you think you got better at regularly taking your statin medication over the course of the study?
        i. [If yes] How did you get better at taking your medication?
        ii. [If yes] Have you been able to maintain this improvement since the study ended? Why or why not?
        iii. [If no] Why do you think your ability to regularly take your statin medication was unchanged by the study?
    c. Did you develop any side effects during the study?
        i. [If yes] What symptoms did you experience, and how did they affect your daily life?
        ii. [If yes] Do you believe they were caused by the statin medication?
            1. [If yes] What gives you the sense that the statins caused these symptoms?
        iii. [If yes] Did you ever stop taking your statin medication or take less as a result of these symptoms?
            1. [If yes] What was the effect, if any, of stopping or taking less?
    d. What have you done, if anything, to help lower your LDL or help you regularly take your medications since the conclusion of the study?

8. *Summative assessments*
    a. What aspect of this trial do you think was most helpful in getting you to regularly take your statin medication, and how?
    b. What aspect of the trial do you think was least helpful, and why?
    c. Did anything else change in your life as a result of the study?

© 2021 Barankay I et al. *JAMA Network Open*.

d. Would you recommend the study to a friend? Why or why not?
e. Would you participate in a similar program if it was offered by your insurer? Why or why not?

9. Is there anything else that you think is important about your experience in this trial that we haven't covered?

10. Thank you so much for your time today! Can you verify your mailing address so that we can send your $50 payment?

1.4. Qualitative Assessment Coding Methods

Transcript coding was managed with NVivo 12 (QSR International). Supervised by JTC, three team members (DP, CP, SC) annotated the same three randomly selected transcripts to generate themes. As a group, the team members discussed these themes to provide further definition and organization into a formal codebook (a taxonomy for thematically categorizing data).[1] A second round of annotation using five more randomly selected transcripts refined the initial codebook. Using this refined codebook, DP and CP participated in two rounds of double coding. In each round, they coded five randomly selected transcripts. Intercoder comparison was performed and kappa values calculated. Any instance of coding that generated a kappa less than 0.6 was discussed and rectified through consensus. After each round, the codebook was revised to refine categories lacking clarity, eliminate those lacking utility, and develop new categories to capture important themes not previously included. Using the final codebook, DP and CP coded all remaining transcripts. All codebook revisions were applied to previously coded transcripts.

[1] MacQueen KM, McLellan E, Kay K, Milstein B. Codebook Development for Team-Based Qualitative Analysis. *CAM Journal.* 1998;10(2):31-36.

© 2021 Barankay I et al. *JAMA Network Open*.
